# Whole genome sequencing reveals a 7 base-pair deletion in *DMD* exon 42 in a dog with muscular dystrophy

**DOI:** 10.1007/s00335-016-9675-2

**Published:** 2016-12-27

**Authors:** Peter P. Nghiem, Luca Bello, Cindy Balog-Alvarez, Sara Mata López, Amanda Bettis, Heather Barnett, Briana Hernandez, Scott J. Schatzberg, Richard J. Piercy, Joe N. Kornegay

**Affiliations:** 1 Department of Veterinary Integrative Biosciences (Mail Stop 4458), College of Veterinary Medicine, Texas A&M University, College Station, TX 77843-4458 USA; 2 Department of Neurosciences, University of Padova, Via Giustiniani 5, 35128 Padova, Italy; 3 Veterinary Emergency and Specialty Center of Santa Fe, 2001 Vivigen Way, Santa Fe, NM 87505 USA; 4 Department of Clinical Sciences and Services, Royal Veterinary College, London, UK

## Abstract

Dystrophin is a key cytoskeletal protein coded by the Duchenne muscular dystrophy (*DMD*) gene located on the X-chromosome. Truncating mutations in the *DMD* gene cause loss of dystrophin and the classical DMD clinical syndrome. Spontaneous *DMD* gene mutations and associated phenotypes occur in several other species. The mdx mouse model and the golden retriever muscular dystrophy (GRMD) canine model have been used extensively to study DMD disease pathogenesis and show efficacy and side effects of putative treatments. Certain *DMD* gene mutations in high-risk, the so-called hot spot areas can be particularly helpful in modeling molecular therapies. Identification of specific mutations has been greatly enhanced by new genomic methods. Whole genome, next generation sequencing (WGS) has been recently used to define DMD patient mutations, but has not been used in dystrophic dogs. A dystrophin-deficient Cavalier King Charles Spaniel (CKCS) dog was evaluated at the functional, histopathological, biochemical, and molecular level. The affected dog’s phenotype was compared to the previously reported canine dystrophinopathies. WGS was then used to detect a 7 base pair deletion in *DMD* exon 42 (c.6051-6057delTCTCAAT mRNA), predicting a frameshift in gene transcription and truncation of dystrophin protein translation. The deletion was confirmed with conventional PCR and Sanger sequencing. This mutation is in a secondary *DMD* gene hotspot area distinct from the one identified earlier at the 5′ donor splice site of intron 50 in the CKCS breed.

**Electronic supplementary material:**

The online version of this article (doi:10.1007/s00335-016-9675-2) contains supplementary material, which is available to authorized users.

## Introduction

Mutations in the Duchenne muscular dystrophy (*DMD*) gene result in decreased to absent dystrophin protein expression (Hoffman et al. 1987). The extensive Leiden database of *DMD* gene mutations showed that 72% of mutations are intragenic deletions of one or more of the 79 exons, with exons 45–53 being the most commonly affected region or hotspot (Aartsma-Rus et al. 2006). Small deletions, insertions, or point mutations account for a further 20% of mutations (Aartsma-Rus et al. 2006). The remaining approximately 7% of *DMD* gene mutations are caused by single or multi-exon duplications, with exons 2–20 being the most commonly affected site (Aartsma-Rus et al. 2006). Most mutations result in a frameshift, while others affect RNA splicing or are nonsense mutations. Becker muscular dystrophy, a less severe dystrophinopathy, is characterized by in-frame *DMD* gene mutations (Aartsma-Rus et al. 2006).

Multiplex ligation-dependent probe amplification (MLPA), a variation of multiplex polymerase chain reaction (PCR) is able to identify deletions and duplications involving one or more exons and is the most widely used molecular technique for diagnosis of DMD (Grimm et al. 2012). When combined with Sanger sequencing of genomic DNA (gDNA) or complementary DNA (cDNA) obtained from retro-transcription of *DMD* messenger RNA, these techniques have a 97.3% sensitivity in detecting *DMD* gene mutations (Grimm et al. 2012). However, sequencing of the entire 12 kb *DMD* coding sequence is time consuming. Next generation sequencing (NGS) of either the whole genome (WGS) or targeted enrichment of the *DMD* gene provides a sensitive and specific molecular technique for DMD diagnosis (Wang et al. 2014; Roucher-Boulez et al. 2015; Okubo et al. 2016). Although the costs are still too high for NGS to be routinely implemented in the dystrophinopathy diagnostic workflow, NGS techniques are becoming more accessible and may soon represent a feasible alternative to the conventional approach for DMD patients and animal models.

Pre-clinical therapeutic development with animal systems is necessary to develop treatments for DMD boys. The prevalence of dystrophin deficiency worldwide for dogs and other non-human species is not known but appears rare based on the small number of reports in the literature. Mammalian models with confirmed *DMD* gene mutations that are used for pre-clinical research include the X-linked muscular dystrophy mouse (mdx) (Bulfield et al. 1984), several canine dystrophinopathies (reviewed in Brinkmeyer-Langford and Kornegay 2013 and summarized in Supplemental Table 1), most notably the golden retriever muscular dystrophy (GRMD) dog (Kornegay et al. 1988); the rat (Larcher et al. 2014); and the pig (Klymiuk et al. 2013). The mdx mouse and GRMD dog are most commonly used for pre-clinical testing of pathogenetic mechanisms and therapeutic development (Kornegay et al. 2014). The clinical syndromes among affected canine breeds are fairly consistent, with generalized muscle weakness, abnormal gait, and dysphagia typically being evident by 3 months and progressing.

Mutations in *DMD* gene hotspot areas are particularly valuable in modeling molecular therapies. A recent report detailed a donor splice site mutation in intron 50 of the *DMD* gene in a group of Cavalier King Charles Spaniel (CKCS) dogs (Walmsley et al. 2010), providing a significant model to test exon skipping treatment strategies. In the CKCS dog studied here, clinical signs similar to other reported canine dystrophinopathies were evident at an early age and progressed. We used WGS in this dog to identify a 7 base pair (bp) deletion in exon 42, a secondary *DMD* gene hot spot area.

## Materials and methods

### Animal Use

The dog was used and cared according to principles outlined in the National Research Council’s Guide for the Care and Use of Laboratory Animals. The dog was first maintained at the University of North Carolina and then at Texas A&M University.

### Microscopy

Methods for light and immunofluorescence microscopy have been described previously (Nghiem et al. 2013). Anti-dystrophin antibodies from Novacastra (Buffalo Grove, IL) were against the rod and C-terminal domains (DYS1 and DYS2) (1:100 dilution, antibodies combined into one experiment). Secondary antibody was goat anti-mouse Alexa Fluor IgG (1:500).

### Western blot

7.5% of SDS–PAGE gels were made with BioRad (Hercules, CA) TGX Stain-Free FastCast Acrylamide Starter Kit. Muscle tissue was ground with a homogenizer at 4 °C in RIPA buffer plus Halt Protease and Phosphatase Single-Use Inhibitor Cocktail (Thermo Scientific; Waltham, MA). Total protein quantification was completed with BioRad’s RC DC Protein Assay. 50 µg of protein was loaded in a gel and electrophoresed for 1 h at 100 V. Proteins were transferred to a PVDF membrane for 1 h at 100 V. Membranes were blocked in 5% bovine serum albumin (BSA) in TBS-T for 4 h. DYS1 and DYS2 were incubated with the PVDF membrane at 1:1,000 dilution in 5% BSA overnight at 4 °C. Sigma–Aldrich’s (St. Louis, MO) desmin antibody (D1033) was used as a loading control (1:1,000). Secondary goat anti-mouse IgG horseradish peroxidase antibody was obtained from Santa Cruz Biotechnology (Dallas, TX) and diluted to 1:5,000 in 5% BSA. Membranes were incubated in SuperSignal West Pico Chemiluminescent Substrate (Thermo Scientific) and exposed to radiographic film. Thermo Scientific Restore Western Blot Stripping Buffer was used to strip the blots.

### PCR and sanger sequencing

To test for presence of the previously reported CKCS intron 50 donor splice site mutation, we used primers developed in the earlier study (Walmsley et al. 2010). Total RNA was isolated from myoblasts extracted from the vastus lateralis (VL) muscle, as detailed previously (Nghiem et al. 2013). Genomic DNA was extracted from muscle tissue with Qiagen DNA Blood and Tissue Kit. Reverse transcriptase (RT)-PCR of total RNA was performed to make cDNA using the Applied Biosystems High Capacity cDNA kit (Foster City, CA), also described previously (Walmsley et al. 2010; Nghiem et al. 2013). PCR was performed with AccuPrime Supermix (Thermo Scientific) on a Thermocycler, under the following conditions: 94 °C for 5 min; 94 °C for 30 s, 58 °C for 30 s, 68 °C for 60 s (cycle repeated 35 times); and 68 °C for 5 min. The PCR product was submitted for Sanger sequencing and restriction fragment length polymorphism (RFLP) mutational analysis, as previously reported (Walmsley et al. 2010). A separate primer design for exons 45–53 can be found in supplemental methods (Supplemental Table 2). PCR was performed with identical conditions, as detailed above.

Nested PCR of gDNA was performed with primers flanking the targeted site at *DMD* exon 42. Outside sense and anti-sense primers were as follows: 5′-GTGGTTTAGGAATTCCACATGTACG-3′ and 5′-TGTCTATACCAGCACACTGTCC-3′, respectively. Inside sense and anti-sense primers were as follows: 5′-CTAAGTCAATCATTGTACTGCG-3′ and 5′-CGATGACTGAAGACATGCCTTTGG-3′, respectively. Reaction products were subjected to gel electrophoresis for quality control. Target bands were cut from the agarose gel and the DNA was extracted and processed. Transformation of the PCR product and cloning into the pCR 2.1 Vector was completed using the Invitrogen TA Cloning Kit, as recommended by the manufacturer. PCR products were submitted to the Texas A&M Gene Technology Lab for Sanger sequencing. Sequence chromatograms were viewed with Sequencher (Gene Codes Corporation; Ann Harbor, MI).

### Whole genome sequencing

Genomic DNA was extracted from muscle using the Qiagen DNA Blood and Tissue Kit (Hilden, Germany). 2 µg of gDNA was prepared and sent to Macrogen (Rockville, MD), where it was clustered and sequenced on the Illumina HiSeq X Ten (San Diego, CA). Briefly, the gDNA was further prepped according to the Illumina TruSeq DNA PCR-free library preparation guide. DNA was fragmented and a final library of 300–400 base pair (bp) average insert size was created. Using an End Repair Mix, the double-stranded DNA 3′ or 5′ overhang fragments were converted into blunt ends, while the 3′–5′ exonuclease removed the 3′ overhangs and the polymerase filled the 5′ overhangs. After end repair, the appropriate library size was selected using Sample Purification Beads. 3′ end adenylation was performed by adding a single ‘A’ nucleotide to the blunted fragments along with a corresponding ‘T’ nucleotide to provide complementary overhang for adapter ligation to the fragment. Multiple indexing adapters were ligated to DNA fragment ends and prepared for flow cell hybridization. Library validation, including a quality control analysis of the sample library and quantification of DNA library templates, was performed by Macrogen. The flow cell containing unique clusters was loaded into the HiSeq X Ten for automated cycles of sequencing by synthesis (extension and imaging). WGS data were aligned with Isaac aligner (Raczy et al. 2013). Isaac Variant Caller was used to identify and genotype single-nucleotide polymorphisms (SNPs) and small insertions/deletions (indels). A variant call format file was produced with the probability of consensus difference from genotype and probability of the called genotype. Mapping quality was performed by Phred-scaled probability of all samples and reported in −log. National Center for Biotechnology Information’s (NCBI) Genome Workbench software (Bethesda, MD) was used to evaluate mutations, SNPs, and indels within the *DMD* gene (compared to the CanFam3.1 whole genome shotgun sequence used as the reference) (Hoeppner et al. 2014).

## Results

### History and physical examination

**Fig. 1:**
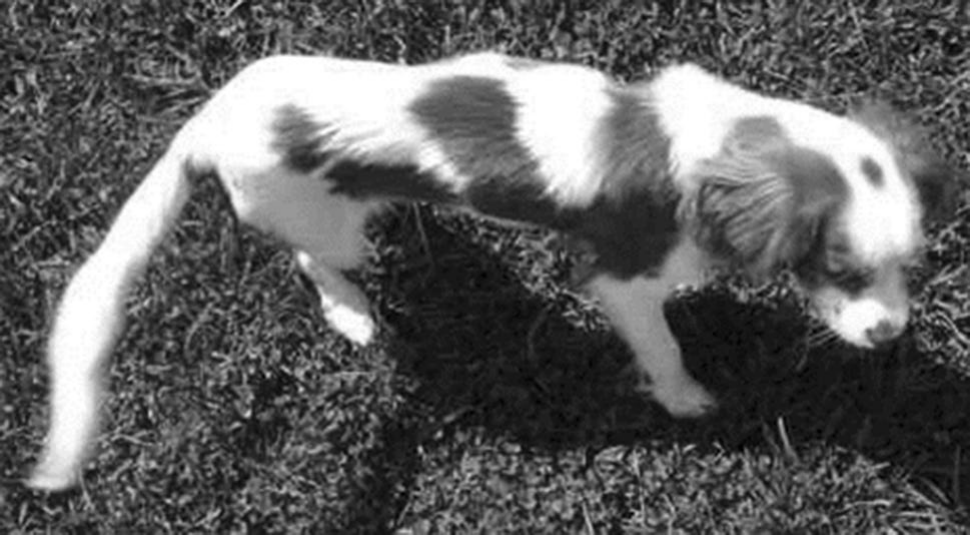
Cavalier King Charles Spaniel dog with dystrophin deficient muscular dystrophy at 6-month-old. Note the generalized skeletal muscle atrophy of the temporal region, limbs and thoracolumbar spine

Clinical syndromes of dogs with confirmed dystrophin deficiency reported in the literature are summarized in Supplemental Table 2. A 6-month-old, intact male CKCS dog (named Buckley) was presented to the University of Georgia Small Animal Teaching Hospital for a 3 months history of dysphagia, ptyalism, nasal congestion, non-progressive coughing, lethargy, and decreased activity compared to other household dogs. The dog was obtained from a CKCS dog breeder at 3 months of age and noted to be smaller and have a weak bark compared to its littermates. It was also unable to properly masticate and swallow dry kibble. After eating, the dog exhibited cough and nasal congestion. Examination revealed generalized skeletal muscle atrophy, macroglossia, ptyalism, nasal discharge, and moderate bilateral carpal hyperextension (Fig. 1). It protracted both pelvic limbs simultaneously during trotting (indicative of a ‘bunny-hopping’ gait), fatigued and subsequently collapsed after trotting for 1–2 min. No other abnormalities were detected on physical examination.

### Biochemical analysis

A complete blood count revealed leukocytosis (white blood cell count of 24,600/µl; normal value [n.v.] 5.5–13,900/µl), with increased numbers of mature neutrophils (16,482/µl; n.v. 2.9–12,000/µl), lymphocytes (5,900/µl; n.v. 0.4-2,900/µl), and eosinophils (1,400/µl; n.v. 0–1,300/µl). A biochemistry panel revealed increased serum activity of creatine kinase (CK; 29,170 U/L; n.v. 63–350 U/L), AST (472 U/L (no reference range available), and ALT 705 U/L; n.v. 12–108 U/L). Concentrations of BUN (8 mg/dl; n.v. 10–30 mg/dl) and creatinine (0.4 mg/dl; n.v. 0.5–1.5 mg/dl) were decreased. Urinalysis was unremarkable. Pre and post-prandial bile acid concentrations, measured to rule out hepatobiliary disease, were also unremarkable.

### Diagnostic imaging

Thoracic radiographs suggested left ventricular enlargement with no evidence of atrial enlargement. A small amount of fluid was seen in the caudal thoracic esophagus. The liver appeared smaller than normal. An echocardiogram study revealed mild systolic and diastolic dysfunction, including prolonged isovolemic contraction and relaxation times. Myocardial wall thickness, contractility, velocity of contractility, and ejection fraction were within normal limits. A cervical ultrasonogram did not reveal abnormalities. The lateral survey projection of a pharyngo-esophogram revealed focal accumulation of gas bubbles in the mid cervical esophagus. Oral pharyngeal bolus formation and esophageal progressive motility were within normal limits on fluoroscopy, but small amounts of liquid barium food mixture consistently accumulated in the cervical esophagus. There was no evidence of achalasia or an esophageal stricture.

Due to the dog’s condition, an inherited muscle disease was suspected, and it was donated for further study. The dog was subsequently transferred to the University of North Carolina-Chapel Hill and then to Texas A&M University. Gradual disease progression was observed over the next 5 years, at which time the dog died due to complications during anesthesia.

### Necropsy and histopathological analysis

**Fig. 2:**
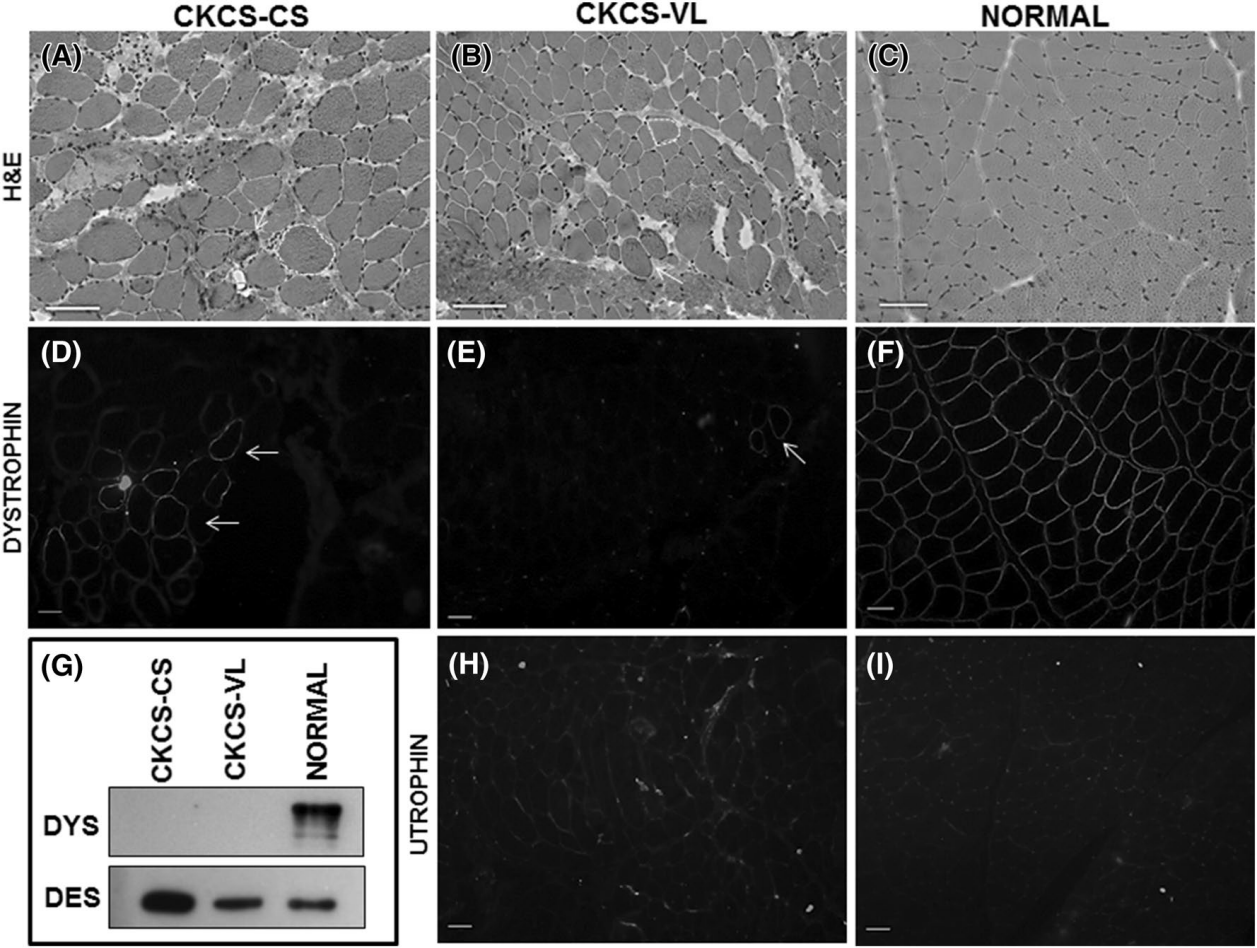
Histopathological and immunofluorescence analyses of dystrophic muscle in the CKCS dog. **a** Cranial sartorius (CS) muscle of the affected CKCS dog showed myofiber size variation with hypertrophy (*white dotted outline*), myofibers with central nuclei indicating regeneration, increased perimyseal connective tissue, inflammation, and myofiber necrosis (*white arrow*). **b** Vastus lateralis (VL) muscle of the CKCS dog showed myofiber size variation (*dotted line*) as a result of degeneration and regeneration, increased perimysial connective tissue, immune cell infiltration, and hypercontracted (hyaline) fibers (*white arrow*). **C**: Normal dog muscle at 6 months of age. Note the uniform size of myofibers, myonuclei at the periphery, and lack of both immune cell infiltration, and increased perimysial connective tissue. **d, e** Dystrophin (DYS1 and DYS2) protein immunostaining of the affected CKCS dog showed a small amount of dystrophin revertant fibers, more so in the CS (**d**) compared to the VL (**e**) muscle (*white arrows*). **f** Normal dog muscle at 6 months showed membranous staining of dystrophin protein. **g** Western blot: Dystrophin (DYS1 and DYS2) was detected in normal muscle, but not in the CS or VL of the affected CKCS. Desmin was used as a loading control. Note that desmin, an intermediate filament that localizes to the sarcolemma, was increased in the hypertrophied, dystrophin-deficient CS muscle. **h** Utrophin protein immunostaining of the CKCS dog showed subtle membranous staining in the CS (not shown) and VL (**h**) muscles with no difference in expression between the muscles. **i** Utrophin protein immunostaining in normal dog muscle was confined to regions consistent with neuromuscular junctions. Marker = 50 µm; ×100 magnification (×10 ocular, ×10 objective)

The dog had variable muscle atrophy and hypertrophy on gross necropsy. Histopathological analysis focused on the cranial sartorius (CS) and vastus lateralis (VL) head of the quadriceps, as these muscles are differentially affected in golden retriever muscular dystrophy (GRMD) dogs (Nghiem et al. 2013). Similar to our previous studies, the VL muscle showed classic dystrophic changes, including variation in myofiber size, myofiber necrosis, inflammation, and increased perimysial connective tissue (Fig. 2b). The CS muscle showed similar dystrophic changes, but consistent with our findings in GRMD (Nghiem et al. 2013), the myofibers were hypertrophied compared to normal and the dystrophic VL muscle (Fig. 2a-c).

### Immunofluorescence microscopy and western blotting

**Fig. 3:**
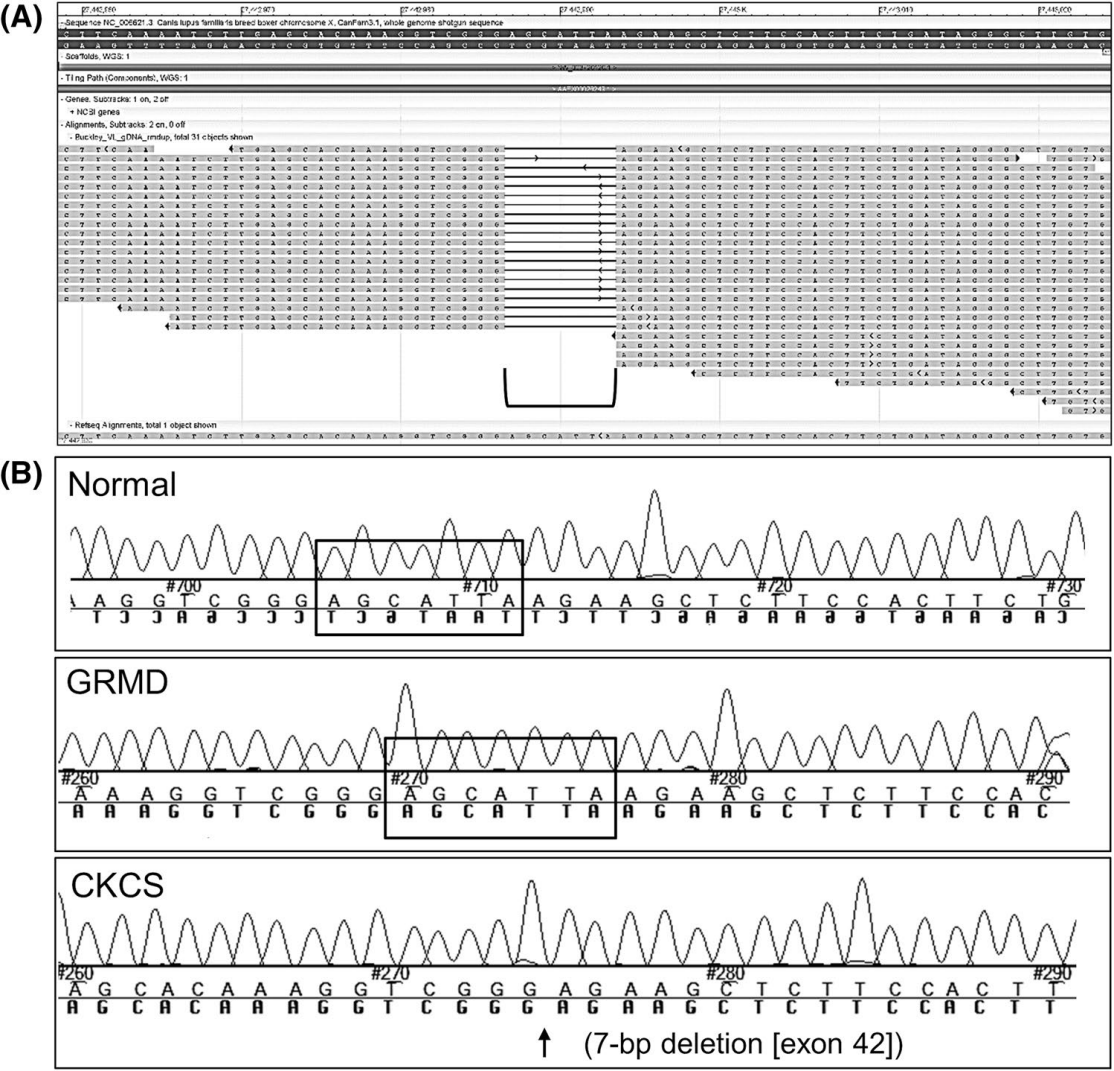
Whole genome sequencing revealed a 7-bp deletion in exon 42 of the *DMD* gene. **a** WGS reads (*bottom* part of image) show the 7-bp deletion (AGCATTA) in exon 42 (bracket). **b** Chromatograms of PCR product Sanger sequencing confirmed the 7-bp deletion in exon 42 of the affected CKCS dog. Normal and the dystrophin-deficient GRMD dog (with splice site mutation in intron 6) revealed the 7-bps were present in exon 42 of the *DMD* gene (*black* box outline)

Together with the dog’s male gender, the elevated serum CK activity and dystrophic skeletal muscle changes were highly suggestive of a dystrophinopathy. Dystrophin protein immunostaining was absent in the CS and VL muscles, except for occasional revertant fibers, particularly in the CS muscle (Fig. 2d, e). Uniform membranous dystrophin staining was seen in a normal 6-month-old dog muscle tissue used as control (3F). Dystrophin was detected in control muscle tissue on western blot, but not in the affected CKCS muscles (Fig. 2g). Desmin, an intermediate filament in skeletal muscle tissue, was used as a protein loading control. Interestingly, desmin appeared to be increased in the hypertrophied CS muscle compared to the dystrophic VL and also normal muscle (Fig. 3g).

Utrophin is homologous with dystrophin and upregulated in dystrophin deficiency, potentially serving as a dystrophin surrogate (Matsumura et al. 1992; Tinsley et al. 1998; Cerletti et al. 2003; Nghiem et al. 2013). We probed CS and VL muscles of the affected dog and found a subtle increase in utrophin at the myofiber membrane, consistent with our previous studies in GRMD dogs (Fig. 2h) (Nghiem et al. 2013). Utrophin expression in control canine muscle, as expected, was localized to neuromuscular junctions and absent in the myofiber membranes (Fig. 2i).

### Molecular analysis of gDNA

A donor splice site mutation (G–T nucleotide mutation) in intron 50 was previously characterized in a group of CKCS dogs (Walmsley et al. 2010). As such, we sought to determine if the CKCS dog in this study had the same mutation. However, the previously characterized splice site mutation in intron 50 was not detected by Sanger sequencing and RFLP (results not shown). To further investigate the causative mutation, we developed primers for the known mutational hotspot at exons 45–53, and performed PCR on extracted gDNA. Agarose gel electrophoretic analysis did not detect a banding pattern suggestive of deletions or duplications compared to a normal dog sample (results not shown).

To continue characterization of the *DMD* gene in this CKCS dog, we performed WGS using the Illumina HiSeq X Ten. The affected sample was compared and referenced to the 2,410,976,875 base-length CanFam3.1 whole genome shotgun sequence (Hoeppner et al. 2014). Mapped sites greater than 1X coverage included 2,374,293,654 bases (98.48% of reference genome) with a total read count of 821,908,096. Mapped reads and bases of 696,176,213 (84.7%) and 99,774,575,483, respectively, were referenced back to CanFam3.1. The mean alignment depth was 41.38. Isaac Variant Caller detected 265,324, 248,521, and 1,518,125 SNPs, insertions and deletions, respectively, which were classified by chromosomes or scaffolds. Duplications were not called.

Evaluation of the entire *DMD* gene (X chromosome, NC_006621.3; 26,290,903..28,444,730) in the CKCS dog revealed 1,007 SNPs and indels (Supplemental Table 3). A 7-bp deletion was detected in exon 42 (positions 27,442,987..27,442,993) (c.6051_6057delTCTCAAT mRNA mutation) (Fig. 3). The deletion predicted a frameshift mutation at amino acid 2017 (leucine) with a stop codon introduced 18 amino acids downstream and premature stoppage of translation (p.Leu2017Leufs*18). PCR of the affected region around the *DMD* exon 42 deletion, followed by Sanger sequencing, were performed to validate the WGS reads. Normal and dystrophin deficient GRMD (splice site mutation in intron 6 of *DMD* gene) (Kornegay et al. 1988; Sharp et al. 1992) samples showed the correct 7-bp in exon 42 (Fig. 3; Supplemental Table 4).

## Discussion

This report details a dystrophin-deficient CKCS with progressive clinical signs until 6 months of age at which time the dog clinically stabilized. This phenotype is in keeping with dystrophin-deficient GRMD dogs perpetuated for research purposes (Valentine et al. 1988). We used WGS to identify a 7-bp deletion in exon 42 of the *DMD* gene, predicting a frameshift mutation and premature halting of translation. This mutation is in a secondary hotspot area distinct from a previously identified splice site mutation in CKCS dogs (Walmsley et al. 2010), thereby providing a potential valuable canine model. Moreover, our study establishes WGS as a viable option compared to conventional PCR and Sanger Sequencing to characterize *DMD* gene mutations in dogs.

The studied CKCS dog presented to the referring veterinarian at 6 months of age with typical historical and clinical signs of muscular dystrophy, including small stature, exercise intolerance, dysphagia, coughing, and a weak bark. Other reported historical and clinical signs in various DMD dog breeds include generalized weakness and other gait abnormalities, muscular atrophy, paradoxical muscle hypertrophy, ptyalism, collapse, with dogs surviving from 3 to 152 months of age (Kornegay et al. 1988; Valentine et al. 1988; Winnand et al. 1994; Schatzberg et al. 1999; Wetterman et al. 2000; Shelton et al. 2001; Bergman et al. 2002; Jones et al. 2004; Wieczorek et al. 2006; Baltzer et al. 2007; Walmsley et al. 2010; Olby et al. 2011; Smith et al. 2011; Beltran et al. 2015; Jenkins et al. 2015). The dog in this report had a clinical course typical of that seen with other canine dystrophinopathies, succumbing at 60 months. On a related note, we are currently conducting molecular studies in a cohort of 52 mildly affected, dystrophin-deficient GRMD dogs that have lived greater than 36 months of age (median = 65 months, 95% CI 55–76 months) (Nghiem, Kornegay, Bello, unpublished data).

Upon finding that the studied dog did not have the CKCS intron 50 mutation, we expanded our analysis to include exons 45 through 53, the known mutation hotspot in DMD patients, but conventional PCR was uninformative. Whole genome sequencing then revealed a 7-bp deletion of exon 42 that was confirmed with PCR and Sanger sequencing. The nonsense mutation predicted a frameshift mutation and a termination of translation. By eliminating steps of selective capture and amplification of specific genomic regions, WGS ensures homogeneous read depth in all genomics regions, increasing sensitivity, even for mutations in coding regions. Moreover, these techniques have become increasingly cost effective, making characterization of novel *DMD* gene mutations in dogs and assessment of pre-clinical molecular trials more practical. While the ~$1,000–2,500 per sample cost of WGS is currently more expensive than conventional PCR and Sanger Sequencing, the time for data generation/interpretation is much improved (~4–8 weeks for data generation; ~4 weeks for interpretation).

A significantly improved canine reference sequence, CanFam3.1, now covers 99.8% of the euchromatic canine genome, making WGS data much easier to analyze, especially in GC rich regions such as promoters and first exons (Hoeppner et al. 2014). On the other hand, because WGS can detect millions of changes in the genome, determining the clinical significance of the many polymorphisms can be challenging. In the studied CKCS dog, we detected over 2 million genomic variants. Further analyses of these other genomic variants are currently underway. We used NCBI’s Genome Browser to analyze the WGS data; other softwares are also available. Finally, certain inbred dogs may show genetic divergence due to outbreeding (to reduce mortality related deaths due to high inbreeding) (Kornegay et al. 2011). This genetic divergence may pose issues for researchers attempting to evaluate genetic causes of disease.

Although not considered part of the main exon 45–53 *DMD* gene hot spot, a recent report identified mutations in exon 42 in 2% of the DMD patient cohort (Barzegar et al. 2015). To our knowledge, this is the first canine dystrophinopathy in which an exon 42 mutation was identified. In theory, if the mutated *DMD* gene transcript was not subjected to nonsense-mediated decay, the studied CKCS dog could have a less severe phenotype. Notably, dystrophin transcript apparently avoids nonsense decay and persists in GRMD dogs (Cotten et al. 2013). Immunofluorescence microscopy revealed a small amount of dystrophin revertant fibers, particularly in the relatively preserved and hypertrophied CS muscle. In contrast, dystrophin was completely absent on immunostaining in the CKCS dogs reported in separate studies by Walmsley et al. and Schatzberg (Walmsley et al. 2010; Schatzberg and Shelton 2004). Dystrophin expression in the CS muscle could occur due to either persistence of protein containing exons 1 through 41 (escaping nonsense-mediated decay) or from alternatively spliced dystrophin mRNA products (Cotten et al. 2013; Schatzberg et al. 1998). This CKCS model could also be valuable in studying molecular therapies such as exon skipping (Aartsma-Rus 2012) or gene editing, targeting exon 42. Similar to many other exons encoding the rod domain of dystrophin, where small pathogenetic mutations often localize, exon 42 is an in-frame exon consisting of a multiple-of-three number of nucleotides (195). Its splicing consensus sequences may be masked by an oligonucleotide at the transcript level (exon skipping), or disabled by gene editing at the genomic level (exon snipping, Kemaladewi and Cohn 2016), to inhibit inclusion of this exon in the mature transcript, and restore the open reading frame of dystrophin. We are evaluating options to perpetuate this canine model.

Because of its similar structure and function, utrophin could act as a dystrophin surrogate to stabilize dystrophic myofiber membranes (Tinsley et al. 1998; Cerletti et al. 2003; Nghiem et al. 2013). Consistent with our previous studies in GRMD dogs (Nghiem et al. 2013), the CKCS dog of this report had a subtle increase in utrophin at the myofiber membrane in both the hypertrophied CS and dystrophic VL muscles. Associated stabilization of the dystrophic membrane could have contributed to this dog living to middle age. However, importantly, mildly affected GRMD dogs used for breeding in our colony routinely live well into adulthood, in keeping with disease amelioration from gene modifiers or other genetic mechanisms. Based on data reported here and in GRMD dogs (Nghiem and Kornegay, unpublished), desmin may contribute to another compensatory pathway in the spared/hypertrophied CS muscle.

In conclusion, the studied CKCS dog had typical, progressive clinical signs of dystrophin deficiency. WGS is a viable diagnostic option to characterize *DMD* gene mutations in dystrophic canines. The 7-bp deletion in exon 42 is analogous with those seen in DMD patients. If perpetuated, this model could have value in studying mechanisms that ameliorate the dystrophic phenotype, to include genetic therapies such as exon skipping and gene editing.

## Electronic supplementary material

Below is the link to the electronic supplementary material.

Supplementary material 1 (DOCX 102 KB)

## References

[CR1] Aartsma-Rus A (2012). Overview on DMD exon skipping. Methods Mol Biol.

[CR2] Aartsma-Rus A (2006). Entries in the Leiden Duchenne muscular dystrophy mutation database: an overview of mutation types and paradoxical cases that confirm the reading-frame rule. Muscle Nerve.

[CR3] Baltzer WI (2007). Dystrophin-deficient muscular dystrophy in a Weimaraner. J Am Anim Hosp Assoc.

[CR4] Barzegar M (2015). Exon Deletion Pattern in Duchene Muscular Dystrophy In North West of Iran. Iran. J Child Neurol.

[CR5] Beltran E (2015). Dystrophin-deficient muscular dystrophy in a Norfolk terrier. J Small Anim Pract.

[CR6] Bergman RL (2002). Dystrophin-deficient muscular dystrophy in a Labrador retriever. J Am Anim Hosp Assoc.

[CR7] Brinkmeyer-Langford C, Kornegay JN (2013). Comparative genomics of X-linked muscular dystrophies: the golden retriever model. Curr Genom.

[CR8] Bulfield G (1984). X chromosome-linked muscular dystrophy (mdx) in the mouse. Proc Natl Acad Sci.

[CR9] Cerletti M (2003). Dystrophic phenotype of canine X-linked muscular dystrophy is mitigated by adenovirus-mediated UTRNophin gene transfer. Gene Ther.

[CR10] Cotten SW (2013). Genetic myostatin decrease in the golden retriever muscular dystrophy model does not significantly affect the ubiquitin proteasome system despite enhancing the severity of disease. Am J Transl Res.

[CR11] Grimm T (2012). Risk assessment and genetic counseling in families with Duchenne muscular dystrophy. Acta Myol.

[CR12] Hoeppner MP (2014). An improved canine genome and a comprehensive catalogue of coding genes and non-coding transcripts. PLoS One.

[CR13] Hoffman EP (1987). Dystrophin: the protein product of the Duchenne muscular dystrophy locus. Cell.

[CR14] Jenkins CA, Forman OP (2015). Identification of a novel frameshift mutation in the DMD gene as the cause of muscular dystrophy in a Norfolk terrier dog. Canine Genet Epidemiol.

[CR15] Jones BR (2004). Muscular dystrophy with truncated dystrophin in a family of Japanese Spitz dogs. J Neurol Sci.

[CR16] Kemaladewi DU, Cohn RD (2016). Exon snipping in Duchenne muscular dystrophy. Trends Mol Med.

[CR17] Klymiuk N (2013). Dystrophin-deficient pigs provide new insights into the hierarchy of physiological derangements of dystrophic muscle. Hum Mol Genet.

[CR18] Kornegay JN (1988). Muscular dystrophy in a litter of golden retriever dogs. Muscle Nerve.

[CR19] Kornegay JN (2011). Golden retriever muscular dystrophy (GRMD): developing and maintaining a colony and physiological functional measurements. Methods Mol Biol.

[CR20] Kornegay JN (2014). Pharmacologic management of Duchenne muscular dystrophy: target identification and preclinical trials. ILAR J.

[CR21] Larcher T (2014). Characterization of dystrophin deficient rats: a new model for Duchenne muscular dystrophy. PLoS One.

[CR22] Matsumura K (1992). Association of dystrophin-related protein with dystrophin-associated proteins in mdx mouse muscle. Nature.

[CR23] Nghiem PP (2013). Sparing of the dystrophin-deficient cranial sartorius muscle is associated with classical and novel hypertrophy pathways in GRMD dogs. Am J Pathol.

[CR24] Okubo M (2016). Genetic diagnosis of Duchenne/Becker muscular dystrophy using next-generation sequencing: validation analysis of DMD mutations. J Hum Genet.

[CR25] Olby NJ (2011). Clinical progression of X-linked muscular dystrophy in two German shorthaired pointers. J Am Vet Med Assoc.

[CR26] Raczy C (2013). Isaac: ultra-fast whole-genome secondary analysis on Illumina sequencing platforms. Bioinformatics.

[CR27] Roucher-Boulez F (2015). A splicing mutation in the DMD gene detected by next-generation sequencing and confirmed by mRNA and protein analysis. Clin Chim Acta.

[CR28] Schatzberg SJ, Shelton GD (2004). Newly identified neuromuscular disorders. Vet Clin North Am Small Anim Pract.

[CR29] Schatzberg SJ (1998). Alternative dystrophin gene transcripts in golden retriever muscular dystrophy. Muscle Nerve.

[CR30] Schatzberg SJ (1999). Molecular analysis of a spontaneous dystrophin ‘knockout’ dog. Neuromuscul Disord.

[CR31] Sharp NJ (1992). An error in dystrophin mRNA processing in golden retriever muscular dystrophy, an animal homologue of Duchenne muscular dystrophy. Genomics.

[CR32] Shelton GD (2001). Muscular dystrophy in female dogs. J Vet Intern Med.

[CR33] Smith BF (2011). An intronic LINE-1 element insertion in the dystrophin gene aborts dystrophin expression and results in Duchenne-like muscular dystrophy in the corgi breed. Lab Invest.

[CR34] Tinsley J (1998). Expression of full-length utrophin prevents muscular dystrophy in mdx mice. Nat Med.

[CR35] Valentine BA (1988). Canine X-linked muscular dystrophy. An animal model of Duchenne muscular dystrophy: clinical studies. J Neurol Sci.

[CR36] Walmsley GL (2010). A duchenne muscular dystrophy gene hot spot mutation in dystrophin-deficient cavalier king charles spaniels is amenable to exon 51 skipping. PLoS One.

[CR37] Wang Y (2014). Whole dystrophin gene analysis by next-generation sequencing: a comprehensive genetic diagnosis of Duchenne and Becker muscular dystrophy. Mol Genet Genom.

[CR38] Wetterman CA (2000). Hypertrophic muscular dystrophy in a young dog. J Am Vet Med Assoc.

[CR39] Wieczorek LA (2006). Dystrophin-deficient muscular dystrophy in an old English sheepdog. Vet Rec.

[CR40] Winnand NJ et al. (1994) Molecular Characterization of severe Duchenne-type muscular dystrophy in a family of Rottweiler dogs. (**Abstract**)

