# Supplementary material for: Whole genome sequencing reveals a 7 base-pair deletion in DMD exon 42 in a dog with muscular dystrophy
Source: Mamm Genome. 2016 Dec 27;28(3):106–13. doi: 10.1007/s00335-016-9675-2 (PMC5371640; doi:10.1007/s00335-016-9675-2)
Supplement: Supplementary file 1 — Supplementary material 1 (DOCX 102 KB) [file 335_2016_9675_MOESM1_ESM.docx]

**Supplemental Table 1: Reported DMD dogs in the literature**. Hx = history; mth = month

| Dog | Signalment | Clinical signs and duration | Physical exam findings | CK  (U/L) | Immunohistochem | Dystrophin Mutational Analysis | Other dx | Cause of death |
| --- | --- | --- | --- | --- | --- | --- | --- | --- |
| Cavalier King Charles Spaniel (Nghiem, Shelton, Piercy) | 1 month (Piercy)  4-5 months (3 dogs)  (Shelton)  6 months, male (Nghiem) | Chronic progressive hx of lethargy, dysphagia, exercise intolerance (Piercy)  Stiff, hunched-up gait, myalgia (Shelton)  3 month hx of dysphagia, cough, nasal congestion, weak bark (Nghiem) | Tetraparesis, poor skeletal muscle mass, reduced spinal reflexes, macroglossia, restricted jaw movement (Piercy)  Generalized skeletal muscle atrophy, macroglossia , bunny-hopping in pelvic limbs, fatigue during gait analysis, mild carpal hyperextension (Nghiem) | 83,000 to 358,000 (myoglobin-uria present (Shelton)  29,010 (Nghiem) | Absent dystrophin (Piercy)  Absent dystrophin (Shelton)  Absent dystrophin (rare revertant fibers) (Nghiem) | 5’ donor splice site mutation in exon 50 (Piercy)  Exon deletion (Shelton)  7-bp deletion in DMD exon 42 (Nghiem) | Prolonged isovolemic contraction & relaxation times, reduced pharyngeal and esophageal motility (Nghiem) | Anesthetic complication (Nghiem) |
| German-Short Haired Pointer (Schatzberg; Olby) | 5 (dog A) and 9 (dog B) months | 3.5 and 6 mth hx of poor growth, muscle atrophy, hind limb weakness & episodic collapse; dog B more severely affected than dog A | Brachygnathism, trismus, wide-based pelvic limb stance, paws laterally rotated, tarsi held close together; concavity of ribs adjacent to the sternum; mild, short-strided gait, fatigue after gait analysis | Dog A: 3,520 - 41,340  Dog B :  2,732-40,200 | Absent dystrophin (dog A and B) | Deletion of entire dystrophin gene | Dog A: hyperechoic myocardial foci (mainly left ventricular free wall, low/normal fractional shortening of the cardiac muscle;  Dog A and B: deep Q waves and increased Q:R ratio | Suspected aspiration pneumonia at 7 years (dog A and B) |
| German Sheppard crossbreed (Shelton) | Puppy | Generalized neuromusc-ular weakness since 6 weeks of age | Severe generalized neuromuscular weakness and exercise intolerance, muscle atrophy of temporal, truncal, and limb mm.; short-strided  and shuffling gait, severe abduction of elbows, bunny  hopping in pelvic limbs; macroglossia, pytalism, pharyngo-esophageal dysmotility, weak bark | 71,000 at 2.5 mths old | Absent dystrophin | Not performed | Not reported | 3 months of age due to recurrent aspiration pneumonia & failure to thrive |
| Golden Retriever (Kornegay) | 1.5 - 2 months, male | Dysphagia, regurgitat-ion, decreased activity level | Progressive, muscle atrophy, joint contractures, decreased activity, walk with stiff, stilted, and shuffling gait with abduction of elbows and bunny  hopping in pelvic limbs, trismus, pytalism, pharyngeal muscle hypertrophy, macroglossia, increased respiratory effort | 16,770 - 24,442 | Absent dystrophin with rare revertant fibers | Splice site mutation in intron 6 | Hyperechoic areas, most obvious in the left ventricular free wall, first  appearing between 6 and 12 months of age; DCM | Variable, neonatal to 152 months due to aspiration pneumonia |
| Japanese Spitz (Jones) | 4-12 mths, male | Slowly progressive generalized neuromus-cular weakness, myalgia, trismus, dysphagia | Pytalism, trismus, muscle atrophy and weakness, nasal discharge and cough, myalgia, stiff gait, fatigue during gait analysis | 330 - > 70,000 | Negative for amino terminal, decreased staining present for carboxy terminal domain of dystrophin (truncated dystrophin); decreased α-SG and γ- SG, increased staining of utrophin | Not reported | Not performed | 15 months due to death or euthanasia (cause unknown) |
| Labrador Retriever (Bergman) | 3.5 months | Generalized neuromuscular  weakness, pytalism, lingual dysmotility and hyper-trophy, and dysphagia, oral food retention | Generalized muscle atrophy,  decreased gag reflex, hypertrophied  muscles of the throat and cranial neck, enlarged  non-mobile tongue | 56,482 | Absence of dystrophin, absence of dystroglycans, small amount of sarcoglycan | Not performed | NAF; fluoroscopic esophogram  unable to swallow liquid barium or  to form a food and barium bolus due to inability to pass food over the tongue | 7 months old due to respiratory difficulties, muscle wasting, and hemato-chezia |
| Miniature Schnauzer  (Shelton) |  | Severe dysphagia | Pytalism,  megaesophagus, decreased gag reflex, mild diffuse neuromuscular  weakness, lingual calcific deposits | Not reported | Absent dystrophin | Not performed | Not reported | Unknown |
| Norfolk Terrier | 6 months (Beltran; Jenkins) | 3-month history of poor growth, reluctant to exercise, progressive and diffuse muscle atrophy | Stiff gait, bunny hopping | 131,106 | Absent dystrophin; increased utrophin and dMHC; reduced α-SG | 1-bp deletion in exon 22 | MRI: hyperintense T2 and short-tau, modest contrast enhancement on T1 | 13 months of age due to progressive disease |
| Old English Sheepdog (Wieczorek) | 3 months | 3 week history of dysphagia, pytalism, mild exercise intolerance  and impaired growth | Lingual and pharyngeal swelling, pytalism, stiff gait, increased muscle tone of both  pelvic limbs, decreased to absent withdrawal-flexor reflexes on all four  limbs, decreased tongue movement, absent gag reflex | 53,396 | Absent dystrophin | Not performed | NAF; Barium swallow revealed poor laryngeal  motility | 3 months old due to progressive disease |
| Pembroke Welsh Corgi  (Smith) | 4.5 mth male | stiffness, apparent muscle enlarge-ment |  | Markedly elevated (results not available) | Absent Dystrophin  Histochemical staining showed involvement of both type 1 and type 2 fibers, although there appeared to be a type 2 fiber loss in some fascicles. There was also fiber type grouping | Not performed  Intronic insertion (Smith and Kornegay) | Not reported | Not reported |
| Rat Terrier  (Wetterman) | 10-mth male | 7 mth hx of progressive pytalism, dysphagia,  abnormal gait, and generalized weakness | Stiff, stilted gait; decreased flexion in stifle, tarsal, and elbow  joints; fatigue during walking; proximal portion of all 4 limbs hypertrophied; severe bilateral  hypertrophy of the sternocephalicus muscles; mild decrease in proprioception of  hind limbs; hypo- to normoreflexive  patellar reflexes;  decreased gag reflex; hypomotile  tongue, reduction in temporalis and masseter muscle mass | 66,420 | Absent rod domain, very few fibers stained to carboxy domain of dystrophin | Not performed | Mildly distended esophagus was evident on  an esophogram obtained after oral liquid barium. The barium was abnormally retained in  the cervical region of the esophagus, indicating  decreased esophageal motility | 4 mths after diagnosis due to progressive cervical muscle hyper-trophy and decreased flexion of the neck |
| Rottweiler (Winnand) | Not reported | Not reported | Severe clinical phenotype, weakness, profound muscle wasting, eventual fixation of hind limbs in extension | Not reported | Absent dystrophin | G to T transversion at nucleotide 8,843 in exon 58, resulting in conversion of glutamate to a stop codon | Not reported | 4 -12 mths for three dogs |
| Weimareiner (Baltzer) | 2 yrs | 1-yr history of  regurgitation, exercise intolerance, and dysphagia | Cryptorchid, poor BCS, generalized muscle atrophy, muscles of the neck,  infraspinatus, and tongue were hypertrophied, intermittent muscle spasms of the neck, short-strided gait | 32,672 | Few dystrophin revertant fibers | Not performed | Hyperechoic regions within left ventricular  free wall, moderate mitral regurgitation | After diagnosis due to severe muscle weakness and poor BCS |

**Supplemental Table 2: Primers for exons 45-53 of the canine *DMD* gene.**

| Exon | Forward (5’ 🡪 3’) | Reverse (5’ 🡪 3’) | PCR product length |
| --- | --- | --- | --- |
| 45 | GACAGCGGCAAAGTGTTGTC | AGCAGCAAAAGCACTGTCTA | 376 |
| 46 | GGAAGAAGCGGATAACGTTGC | TGGACATGTCAGTCAGCCAG | 656 |
| 47 | CCTGCGCCAGGGAATTCTAA | TCCACTGAAGATTTGTCTGCT | 125 |
| 48 | TCACTCTTTTTCCTTGCAGGT | AGTGAAAAGTCCCTACCTTGATGT | 200 |
| 49 | AGTATGTTCCGCAATGGCCT | TTACCTTCGCTGGCTGAGTG | 287 |
| 51a | GAAATCCTTCAGTGCCAATGTA | ATTCCTCAGGTTCTCTGGGC | 447 |
| 51b | ACCTGCACTGGCAGATTTCA | GGAGGAAAATTGGCACAGGC | 569 |
| 52 | GCAACGCTGCAGGATTTGGA | GGCCTCATTCCTGAGGATAGC | 791 |
| 53 | AAGGAGGCTCCCTACACAGT | CATGTAGCTCAGCACAGCCT | 940 |

**Supplemental Table 3: Analysis of the DMD gene with WGS.** 1,007 variants detected.

| Position | Incorrect base(s) | Correct base(s) according to CanFam3.1 | Note |
| --- | --- | --- | --- |
| 27005339 | G | C |  |
| 27004841 | C | T |  |
| 27004123 | C | A |  |
| 27004090 |  |  | Insertion of a base |
| 27004386 | T | A | 5 every other bases |
| 27004379 | C | T |  |
| 27266813 | C | G |  |
| 27267717 |  |  | Insertion of a base |
| 27267764 | ACACAC | GAGACA |  |
| 27268025 | T | C |  |
| 27268075 | G | A |  |
| 27268188 |  |  | Insertion of a base |
| 27268194 | T | A |  |
| 27269205 | G | A |  |
| 27271132 | T | C |  |
| 27272420 | T | C |  |
| 27272895 | GA | TT |  |
| 27279563 |  |  | Insertion of a base |
| 27280942 | T | C |  |
| 27283011 |  |  | Insertion of a base |
| 27290530 | T | A |  |
| 27297757 | A | G |  |
| 27297852 | GG | GT |  |
| 27301376 | T | C |  |
| 27304394 |  |  | Insertion of a base |
| 27310841 | G | A |  |
| 27311268 | C | T |  |
| 27315032 | TT | GC |  |
| 27315229 | T | G |  |
| 27315246 | C | T |  |
| 27315400 | G | A |  |
| 27317197 |  |  | Insertion of a base |
| 27320383 | C | T |  |
| 27320618 | G | A |  |
| 27322509 | A | G |  |
| 27323182 | A | T |  |
| 27323298 |  |  | Insertion of a base |
| 27323492 | C | T |  |
| 27323817 |  |  | Insertion of a base |
| 27324607 | T | C |  |
| 27325971 |  |  | Insertion of a base |
| 27326030 | G | T |  |
| 27327391 | C | T |  |
| 27328463 |  |  | Insertion of a base |
| 27330038 | A | T |  |
| 27330081 | TATATATAT | GAGAGAGAG | Every other base |
| 27332392 | T | C |  |
| 27335457 | T | C |  |
| 27335661 | G | C |  |
| 27336879 |  |  | Insertion of a base |
| 27338807 | T | G |  |
| 27341874 | T | C |  |
| 27343263 | A | G |  |
| 27343345 | C | A |  |
| 27343540 | C | T |  |
| 27349088 | G | T |  |
| 27350380 | T | C |  |
| 27350640 | A | G |  |
| 27351047 | C | T |  |
| 27351100 |  |  | Insertion of a base |
| 27357310 | G | C |  |
| 27357314 | G | T |  |
| 27357353 | AAA | TTT |  |
| 27357357 | A | T |  |
| 27357668 | T | C |  |
| 27358055 | A | G |  |
| 27360190 | T | G |  |
| 27361965 | G | T |  |
| 27362312 | G | A |  |
| 27365845 | A | G |  |
| 27367016 | G | A |  |
| 27367181 | T | G |  |
| 27367290 | G | C |  |
| 27367928 | G | A |  |
| 27375709 |  |  | Insertion of a base |
| 27380394 | T | A |  |
| 27387011 |  |  | Insertion of a base |
| 27394750 | T | A |  |
| 27399045 | T | G |  |
| 27403436 |  |  | Insertion of a base |
| 27403620 | G | C |  |
| 27407840 | T | G |  |
| 27408103 | T | A |  |
| 27410572 | T | C |  |
| 27411562 | T | C |  |
| 27412125 |  |  | Insertion of a base |
| 27413000 | A | G |  |
| 27413950 | T | C |  |
| 27415064 | A | G |  |
| 27415202 | C | T |  |
| 27416607 | G | A |  |
| 27417361 | T | C |  |
| 27419247 |  |  | Insertion of a base |
| 27420556 | A | G |  |
| 27425888 | G | T |  |
| 27426005 | G | T |  |
| 27426886 |  |  | Insertion of a base |
| 27427019 |  |  | Insertion of a base |
| 27428141 | T | C |  |
| 27429162 | C | G |  |
| 27429944 |  |  | Insertion of a base |
| 27430445 | T | C |  |
| 27433152 |  |  | Insertion of a base |
| 27437135 | T | G |  |
| 27437349 |  |  | Insertion of a base |
| 27440205 | A | G |  |
| 27440214 | T | C |  |
| 27442987 | NONE | AGCATTA |  |
| 27443861 | C | T |  |
| 27446745 | C | T |  |
| 27448310 |  |  | Insertion of a base |
| 27448603 |  |  | Insertion of a base |
| 27450842 | C | G |  |
| 27452307 | C | T | Forward and reverse arrows |
| 27453084 | G | A |  |
| 27453308 | AT | GA |  |
| 27455534 | A | T |  |
| 27456691 | T | C |  |
| 27456749 | C | T |  |
| 27457031 | G | A |  |
| 27459602 | C | A |  |
| 27459959 | C | T |  |
| 27464170 | G | A |  |
| 27465643 | A | G |  |
| 27465647 | A | G |  |
| 27467728 | C | A |  |
| 27470351 | A | T |  |
| 27470983 | A | G |  |
| 27472344 | T | A |  |
| 27473797 |  |  | Insertion of a base |
| 27474939 | C | A |  |
| 27479994 | A | G |  |
| 27481322 | G | A |  |
| 27481337 | C | T |  |
| 27481431 | C | T |  |
| 27487859 | A | G |  |
| 27488015 | T | A |  |
| 27488139 | T | C |  |
| 27488361 | NONE | ATTC |  |
| 27489287 | C | T |  |
| 27490015 | A | G |  |
| 27490144 |  |  | Insertion of a base |
| 27491213 |  |  | Insertion of a base |
| 27491438 | C | T |  |
| 27492276 | T | C |  |
| 27492291 | T | G |  |
| 27493422 | C | T |  |
| 27494966-27495145 |  |  |  |
| 27495302 | T | C |  |
| 27496532 | A | G |  |
| 27496754 | A | T |  |
| 27497428 | T | C |  |
| 27500711 | C | A |  |
| 27501226 | T | G |  |
| 27501247 | G | A |  |
| 27503327 | C | G |  |
| 27505009 | G | A |  |
| 27507904 | C | T |  |
| 27509361 | A | G |  |
| 27510545 | T | C |  |
| 27511094 | A | G |  |
| 27511525 |  |  | Insertion of a base |
| 27512715 | C | T |  |
| 27513287 |  |  | Insertion of a base |
| 27513597 |  |  | Insertion of a base |
| 27515727 | NONE | AA |  |
| 27515970 | C | T |  |
| 27517878 | A | T |  |
| 27520627 | G | A |  |
| 27521789 | T | C |  |
| 27525284 | A | G |  |
| 27526079 | A | C |  |
| 27526172 | C | A |  |
| 27526467 |  |  | Insertion of a base |
| 27526811 |  |  | Insertion of a base |
| 27527343 |  |  | Insertion of a base |
| 27529349 | T | C |  |
| 27530914 | A | G |  |
| 27532030 | G | A |  |
| 27533062 |  |  | Insertion of a base |
| 27533623 | T | C |  |
| 27533885 |  |  | Insertion of a base |
| 27534523 | T | C |  |
| 27535976 | C | A |  |
| 27537953 |  |  | Insertion of a base |
| 27538944 |  |  | Insertion of a base |
| 27539050 | A | C |  |
| 27540150 | T | C |  |
| 27543489 | C | T |  |
| 27543696 | T | C |  |
| 27544305 | G | A |  |
| 27547709 | G | A |  |
| 27547836 | C | T |  |
| 27554263 | A | G |  |
| 27555106 | A | G |  |
| 27556480 | G | A |  |
| 27557918 |  |  | Insertion of a base |
| 27558791 |  |  | Insertion of a base |
| 27561039 | T | G |  |
| 27562003 | T | G |  |
| 27563187 | C | T |  |
| 27563704 | T | C |  |
| 27563917 | NONE | A |  |
| 27566416-27566608 |  |  |  |
| 27567309 | A | G |  |
| 27568108 | G | A |  |
| 27568334 | T | A |  |
| 27569533 |  |  | Insertion of a base |
| 27570387 | C | A |  |
| 27570655 | G | A |  |
| 27571421 | G | A |  |
| 27571785 | T | C |  |
| 27571790 |  |  | Insertion of a base |
| 27573150 | T | C |  |
| 27573174 | A | G |  |
| 27574175 | T | G |  |
| 27580784 | ACA | TCT |  |
| 27583993 |  |  | Insertion of a base |
| 27586803 | T | C |  |
| 27587961 | AAA | TAT |  |
| 27591255 | A | G |  |
| 27595163 |  |  | Insertion of a base |
| 27595781 | A | G |  |
| 27597502 | A | T |  |
| 27605053 | C | G |  |
| 27606590 | A | G |  |
| 27608210 |  |  | Insertion of a base |
| 27614746 | A | T |  |
| 27614749 | A | G |  |
| 27614481 |  |  | None |
| 27614492 | A | G |  |
| 27619041 | C | T |  |
| 27620787 | ANY OTHER | T |  |
| 27622466 | A | G |  |
| 27624904 | A | T |  |
| 27628212 | T | A |  |
| 27630533 | C | T |  |
| 27630585 | A | T |  |
| 27635674 | G | C |  |
| 27638634 | CCCCC | ACACA |  |
| 27638647 | CAA | AAC |  |
| 27644676 | T | C |  |
| 27645126 | A | C |  |
| 27645934 | T | C |  |
| 27648055 | G | A |  |
| 27648489 |  |  | Insertion of a base |
| 27649143 | A | G |  |
| 27649324 |  |  | Insertion of a base |
| 27649553 | T | C |  |
| 27649603 | C | T |  |
| 27653593 |  |  | Insertion of a base |
| 27653618 |  |  | Insertion of a base |
| 27654333 | A | G |  |
| 27655632 | C | G |  |
| 27655966 | T | C |  |
| 27656471 | A | G |  |
| 27656490 | C | T |  |
| 27656700 | T | C |  |
| 27656710-27656941 |  |  | Missing sequence |
| 27657222 | G | T |  |
| 27657456 | G | C |  |
| 27661150 | T | C |  |
| 27663487 | A | T |  |
| 27664145 |  |  | Insertion of a base |
| 27664402 | C | T |  |
| 27664505 | A | T |  |
| 27665130 | G | A |  |
| 27665882 | G | A |  |
| 27668284 | C | T |  |
| 27668963 | A | G |  |
| 27672220 | T | C |  |
| 27673400 | C | T |  |
| 27674694 | G | A |  |
| 27677655 | G | A |  |
| 27681587 |  |  | Insertion of a base |
| 27681617 | A | T |  |
| 27681925 | A | C |  |
| 27682951 | A | T |  |
| 27683462 | A | G |  |
| 27684865 | T | C |  |
| 27687045 | C | A |  |
| 27689558 | C | T |  |
| 27691044 | T | G |  |
| 27691850 |  |  | Insertion of a base |
| 27691939 | G | T |  |
| 27692769 | A | G |  |
| 27694018 |  |  | Insertion of a base |
| 27695382 | G | A |  |
| 27697795 | A | G |  |
| 27700630 | T | C |  |
| 27701012 | A | T |  |
| 27702484 | A | C |  |
| 27702512 | G | C |  |
| 27703265 | G | A |  |
| 27705002 | A | G |  |
| 27708165 | C | T |  |
| 27711474 | T | C |  |
| 27713220 |  |  | Insertion of a base |
| 27715487 |  |  | Insertion of a base |
| 27721899 | T | A |  |
| 27728738 | A | C |  |
| 27732840 | A | G |  |
| 27739211 | C | T |  |
| 27740076 | A | G |  |
| 27747252 | G | A |  |
| 27748289 | C | T |  |
| 27750461 | C | T |  |
| 27750577 | T | C |  |
| 27750883 | G | A |  |
| 27751261 | C | T |  |
| 27752391 | A | G |  |
| 27752659 |  |  | Insertion of a base |
| 27755281 | AG | CA |  |
| 27755293 | A | G |  |
| 27755498 | A | G |  |
| 27756764 | A | G |  |
| 27759008 | NONE | A |  |
| 27762698 | C | T |  |
| 27765504 | C | T |  |
| 27765659 | T | C |  |
| 27769152 | A | T |  |
| 27769439 | G | A |  |
| 27772769 | NONE | TGGA |  |
| 27773287 |  |  | Insertion of a base |
| 27773522 | NONE | G |  |
| 27777600 | A | T |  |
| 27777637 | T | C |  |
| 27779790 |  |  | Insertion of a base |
| 277809430 |  |  | Insertion of a base |
| 27782933 | NONE |  | Approximately 1000bp missing in all reads |
| 27785688 |  |  | Approximately 1000bp missing in all reads |
| 27791124 | C | T |  |
| 27791393 | NONE | AT |  |
| 27791644 | NONE | G |  |
| 27792384 | A | G |  |
| 27794306 | C | G |  |
| 27794793 | G | T |  |
| 27795102 | NONE | TAAAATAAAAT MISSING, APPROX 600 BP |  |
| 27796060 | A | G |  |
| 27796214 | T | C |  |
| 27796901 | T | C |  |
| 27797778 | T | C |  |
| 27797993 | G | A |  |
| 27800892 | G | T |  |
| 27800907 | G | A |  |
| 27801177 | G | A |  |
| 27801588 | NONE | A |  |
| 27803371 | A | G |  |
| 27803458 | C | A |  |
| 27804844 |  |  | Insertion of a base |
| 27804428 | NONE | TA |  |
| 27804462 |  |  | Insertion of a base |
| 27805582 |  |  | Insertion of a base |
| 27806580 | T | A |  |
| 27808505 | T | C |  |
| 27808584 | C | T |  |
| 27808635 |  |  | Insertion of a base |
| 27808668 | A | T |  |
| 27808676 | A | T |  |
| 27809027 | C | T |  |
| 27809070 | A | T |  |
| 27810636 |  |  | Insertion of a base |
| 27811138 | T | C |  |
| 27812668 | TT | GC |  |
| 27812899 | NONE | A |  |
| 27814656 | C | T |  |
| 27815084 | C | T |  |
| 27815517 | A | G |  |
| 27817341 | C | G |  |
| 27818679 | C | T |  |
| 27821953 |  |  | Insertion of a base |
| 27822447 | C | T |  |
| 27824138 | A | C |  |
| 27824141 | C | T |  |
| 27824491 | NONE | A |  |
| 27824737 | C | G |  |
| 27825698 | C | T |  |
| 27829336 | NONE | A |  |
| 27830248 | G | T |  |
| 27830631 | A | G |  |
| 27831821 |  |  | Insertion of a base |
| 27832109 |  |  | Insertion of a base |
| 27832561 | A | G |  |
| 27832568 | NONE | A |  |
| 27832570 | C | T |  |
| 27832979 |  |  | Insertion of a base |
| 27834226 | G | C |  |
| 27834511 | A | G |  |
| 27834604 | G | T |  |
| 27834631 | G | C |  |
| 27834660 | T | A |  |
| 27837546 |  |  | Insertion of base |
| 27838736 |  |  | Insertion of base |
| 27838801 | T | C |  |
| 27842002 | G | T |  |
| 27842075 | C | T |  |
| 27842192 | A | T |  |
| 27842271 | A | T |  |
| 27843057 | G | T |  |
| 27843085 | A | T |  |
| 27845268 | C | T |  |
| 27845715 |  |  | Insertion of base |
| 27847120 | T | C |  |
| 27848520 | NONE | TGGCTTA |  |
| 27848539 | A | G |  |
| 27849144 | G | A |  |
| 27850356 | G | T |  |
| 27850527 | G | A |  |
| 27852136 |  |  | Insertion of base |
| 27853361 | C | G |  |
| 27853780 |  |  | Insertion of base |
| 27854168 | T | C |  |
| 27854462 |  |  | Insertion of base |
| 27855361 | T | C |  |
| 27856415 | NONE | C |  |
| 27856433 | T | C |  |
| 27857326 | T | A |  |
| 27857543 | G | A |  |
| 27858846 | NONE | AAGA |  |
| 27858995 | C | G |  |
| 27859601 | AGAG | GAGA |  |
| 27859771 | NONE | G |  |
| 27859846 | C | T |  |
| 27861979 | A | T |  |
| 27861990 | A | C |  |
| 27861999 | A | T |  |
| 27862034 | AG | TA |  |
| 27862051 | AAA | GAC |  |
| 27862060 | GGA | AGT |  |
| 27862066 | TCA | ATC |  |
| 27862075 | NONE | AGCGGTAG |  |
| 27862118 |  |  | Insertion of base |
| 27862125 | G | A |  |
| 27864286 |  |  | Insertion of base |
| 27864320 |  |  | Insertion of base |
| 27864322 | C | G |  |
| 27865825 |  |  | Insertion of base |
| 27865869 |  |  | Insertion of base |
| 27865868 |  |  | Insertion of base |
| 27866112 | C | T |  |
| 27868361 |  |  | Insertion of base |
| 27868460 | T | C |  |
| 27871504 |  |  | Insertion of base |
| 27871536 | G | C |  |
| 27871824 | T | A |  |
| 27872793 |  |  | Insertion of base |
| 27875750 | C | T |  |
| 27877400 | NONE | APPROX 400 BP DELETION |  |
| 27878800 | NONE | APPROX 400 BP DELETION |  |
| 27880600 | NONE | APPROX 200 BP DELETION |  |
| 27882497 | A | C |  |
| 27882515 | A | G |  |
| 27882553 | G | A |  |
| 27882687 |  |  | Insertion of base |
| 27883656 | T | G |  |
| 27883899 |  |  | Insertion of base |
| 27884560 | A | C |  |
| 27886281 | A | G |  |
| 27888110 | C | T |  |
| 27888735 |  |  | Insertion of base |
| 27888866 | T | G |  |
| 27889483 | NONE | AA |  |
| 27891433 | A | G |  |
| 27891722-776 | NONE | NONE | Reference genome and CKCS missing area |
| 27892542 | T | A |  |
| 27894642 |  |  | Insertion of base |
| 27894971 | G | C |  |
| 27895024 | G | A |  |
| 27896531 |  |  | Insertion of base |
| 27897429 | A | G |  |
| 27897881 | A | G |  |
| 27898293 |  |  | Insertion of base |
| 27898701 | T | C |  |
| 27898734 | G | A |  |
| 27899102 | G | A |  |
| 27899492 |  |  | Insertion of base |
| 27899635 | T | C |  |
| 27899751 | T | A |  |
| 27902229 | T | G |  |
| 27902500 |  |  | Insertion of base |
| 27904538 |  |  | Insertion of base |
| 27904873 | C | T |  |
| 27905937 |  |  | Insertion of base |
| 27906898 | NONE | C |  |
| 27907196 | NONE | TTT |  |
| 27907313 |  |  |  |
| 27909349 |  |  | Insertion of base |
| 27909956 |  |  | Insertion of base |
| 27910212 | A | G |  |
| 27910952 |  |  | Insertion of base |
| 27912050 | G | A |  |
| 27915312 | T | G |  |
| 27916209 | C | T |  |
| 27916595 | A | G |  |
| 27916709 |  |  | Insertion of base |
| 27916803 |  |  | Insertion of base |
| 27917521 | NONE | TAGTTGTAGAGTGTTGTGTCTCTTTTGTTTGC |  |
| 27917696 |  |  | Insertion of base |
| 27918801 | A | G |  |
| 27919232 | T | A |  |
| 27920748 | C | T |  |
| 27921137 | A | C |  |
| 27921542 | NONE | TCT |  |
| 27922220 |  |  | Insertion of base |
| 27922609 | G | T |  |
| 27925739 | C | G |  |
| 27925824 | C | T |  |
| 27926685 |  |  | Insertion of a base |
| 27930056 | A | G |  |
| 27931193 | TA | AT |  |
| 27931215 |  |  | Insertion of a base |
| 27932177 | G | A |  |
| 27934142 | C | T |  |
| 27934506 | G | A |  |
| 27934585 | A | C |  |
| 27934967 | G | A |  |
| 27934984 |  |  | Insertion of a base |
| 27935529 | G | A |  |
| 27935785 | G | A |  |
| 27937691 | C | T |  |
| 27941250 | NONE | MISSING > 1000BP |  |
| 27942165 | G | A |  |
| 27942429 | NONE | T |  |
| 27946258 | A | G |  |
| 27946524 |  |  | Insertion of a base |
| 27946557 | C | G |  |
| 27947206 | T | C |  |
| 27947230 | C | T |  |
| 27948491 | NONE | MISSING BPS UNTIL 27948705 |  |
| 27949788 | A | G |  |
| 27949793 | T | C |  |
| 27950093 | T | G |  |
| 27951307 |  |  | Insertion of a base |
| 27953222 | T | C |  |
| 27953292 | T | A |  |
| 27955239 | T | C |  |
| 27959781 |  |  | Insertion of a base |
| 27970066 | G | C |  |
| 27971404 | C | T |  |
| 27971524 | T | C |  |
| 27971720 | T | C |  |
| 27971808 | T | C |  |
| 27971975 | A | G |  |
| 27972056 | G | A |  |
| 27972067 | C | A |  |
| 27972358 | G | A |  |
| 27972843 | A | G |  |
| 27973656 | NONE | MISSING BP UNTIL 27973909 |  |
| 27977023 | NONE | TT |  |
| 27977708 | NONE | AA |  |
| 27979136 |  |  | Insertion of a base |
| 27979244 | T | C |  |
| 27979787 | G | A |  |
| 27982183 | A | G |  |
| 27984400 | A | G |  |
| 27989055 | T | C |  |
| 27989059 | A | G |  |
| 27989063 | T | A |  |
| 27989067 | G | T |  |
| 27989068 | T | A |  |
| 27989071 | C | T |  |
| 27989073 | T | G |  |
| 27989079 | C | G |  |
| 27989088 | A | C |  |
| 27989116 | C | G |  |
| 27989148 | T | C |  |
| 27994811 | T | C |  |
| 27995433 | NONE | A |  |
| 27995918 | A | C |  |
| 27999507 |  |  | Insertion of a base |
| 28005926 | C | T |  |
| 28006165 | G | A |  |
| 28009300 | A | G |  |
| 28009941 | C | T |  |
| 28009999 | NONE | A |  |
| 28010109 | A | G |  |
| 28011898 | A | G |  |
| 28015535 |  |  | Insertion of a base |
| 28015524 |  |  | Insertion of a base |
| 28017436 | NONE | TGGTTATTTAGCCAAATAACCTTGAATAA |  |
| 28017537 | G | T |  |
| 28018073 |  |  | Insertion of a base |
| 28018766 | NONE | C |  |
| 28018768 | T | A |  |
| 28022062 | A | G |  |
| 28023293 | G | A |  |
| 28025438 | T | A |  |
| 28029427 | G | A |  |
| 28030970 | T | C |  |
| 28035758 | T | G |  |
| 28036030 | T | C |  |
| 28036522 |  |  | Insertion of a base |
| 28036539 | A | G |  |
| 28036981 | G | A |  |
| 28037253 | G | C |  |
| 28038671 | NONE | CTC |  |
| 28038834 | A | G |  |
| 28039371 |  |  | Insertion of a base |
| 28040605 | A | G |  |
| 28040940 | G | A |  |
| 28040963 | G | T |  |
| 28040963 | G | T |  |
| 28042662 | NONE | AATA |  |
| 28043890 |  |  | Insertion of a base |
| 28044153 |  |  | Insertion of a base |
| 28046569 |  |  | Insertion of a base |
| 28055983 | A | G |  |
| 28058084 | AAATCAT | CCAATCA |  |
| 28059380 | A | G |  |
| 28061939 | T | C |  |
| 28062366 | C | G |  |
| 28063353 | T | C |  |
| 28063503 | A | C |  |
| 28064412 | G | A |  |
| 28066560 | G | A |  |
| 28067664 | T | A |  |
| 28067863 | G | A |  |
| 28068184 | C | T |  |
| 28068948 | TCTCT | ACATC |  |
| 28070006 | T | C |  |
| 28073152 | NONE | T |  |
| 28073753 | NONE | GTGT |  |
| 28074525 | G | A |  |
| 28075487 | T | C |  |
| 28075531 | G | T |  |
| 28076497 | G | A |  |
| 28077005 | G | A |  |
| 28077251 |  |  | Insertion of a base |
| 28078333 | T | C |  |
| 28083230 | NONE | T |  |
| 28083839 | G | C |  |
| 28083943 | CT | TG |  |
| 28087036 |  |  | Insertion of a base |
| 28087537 |  |  | Insertion of a base |
| 28087697 | A | C |  |
| 28089120 |  |  | Insertion of a base |
| 28089517 | A | G |  |
| 28091135 | G | A |  |
| 28092093 | NONE | C |  |
| 28092196 | T | C |  |
| 28092712 | G | A |  |
| 28094028 |  |  | Insertion of a base |
| 28094094 | C | T |  |
| 28094520 | G | A |  |
| 28103707 | C | A |  |
| 28104214 | NONE | T |  |
| 28104214 | C | T |  |
| 28104218 | C | T |  |
| 28105116 | A | C |  |
| 28106792 | C | T |  |
| 28106944 | G | T |  |
| 28107022 | T | C |  |
| 28107037 |  |  | Insertion of a base |
| 28107068 | T | C |  |
| 28107333 | A | T |  |
| 28107489 | G | A |  |
| 28107750 | G | A |  |
| 28107913 | A | G |  |
| 28107964 | C | T |  |
| 28110115 |  |  | Insertion of a base |
| 28110369 | G | A |  |
| 28110398 | T | A |  |
| 28110764 | NONE | TTTTT |  |
| 28112377 | A | G |  |
| 28113481 | NONE | GAGAGAGA |  |
| 28114089 | T | G |  |
| 28114109 | A | T |  |
| 28117179 | T | C |  |
| 28118053 | G | A |  |
| 28119327 | G | A |  |
| 28119425 | T | G |  |
| 28121007 | NONE | A |  |
| 28121093 | G | A |  |
| 28123737 | C | G |  |
| 28123959 | G | C |  |
| 28124751 | G | A |  |
| 28125520 | NONE | GA |  |
| 28125664 | T | G |  |
| 28127849 | T | A |  |
| 28127975 | C | T |  |
| 28130306 |  |  | Insertion of a base |
| 28138801 | NONE | A |  |
| 28142018 | A | C |  |
| 28143439 |  |  | Insertion of a base |
| 28143472 | NONE | T |  |
| 28146808 | ATAG | TGAT |  |
| 28146814 | NONE | A |  |
| 28147278 | G | C |  |
| 28149080 | ATTTTTAAT | TTTATTTTA |  |
| 28150783 | NONE | DELETION UNTIL 28151006 |  |
| 28151012 | ACA | CCG |  |
| 28152999 | G | A |  |
| 28154934 |  |  | Insertion of a base |
| 28156120 | C | T | Close to exon |
| 28156406 | T | C |  |
| 28156724 | T | C |  |
| 28157006 | A | G |  |
| 28158362 | G | A |  |
| 28158981 | G | C |  |
| 28159907 | TG | CA |  |
| 28159049 | C | T |  |
| 28159516 | G | A |  |
| 28159604 | T | C |  |
| 28160033 | G | C |  |
| 28160084 | C | G |  |
| 28160220 | G | C |  |
| 28160231 | T | C |  |
| 28160243 | NONE | CA |  |
| 28160663 | T | C |  |
| 28160830 | G | T |  |
| 28160856 | G | C |  |
| 28161820 | A | T |  |
| 28162190 | A | G |  |
| 28162586 | G | A |  |
| 28162675 | G | A |  |
| 28162694 | C | T |  |
| 28163817 | T | C |  |
| 28164134 | A | G |  |
| 28164212 | A | G |  |
| 28164217 | C | G |  |
| 28164229 | C | T |  |
| 28164236 | C | A |  |
| 28164240 | T | A |  |
| 28164251 | G | A |  |
| 28164256 | C | T |  |
| 28164263 | C | G |  |
| 28164269 | AACG | GATA |  |
| 28164279 | TCCAACAGGGT | AATGACAGGAC |  |
| 28164298 | CCCAG | TGCAA |  |
| 28164461 | A | G |  |
| 28164581 | G | A |  |
| 28164940 |  |  | Insertion of a base |
| 28165026 | A | G |  |
| 28165977 | A | G |  |
| 28166026 | NONE | T |  |
| 28166093 | GC | AT |  |
| 28166366 | G | C |  |
| 28166385 | A | C |  |
| 28167664 | T | C |  |
| 28167787 | NONE | GAACTCACCATGG |  |
| 28168668 | T | G |  |
| 28168932 | C | T |  |
| 28169069 |  |  | Insertion of a base |
| 28169076 | T | C |  |
| 28169213 | GTC | CTA |  |
| 28169315 | NONE | T |  |
| 28169341 | G | A |  |
| 28169448 | NONE | TGTGTGTGTG |  |
| 28170275 | NONE | AA |  |
| 28170556 | A | G |  |
| 28170920 | G | T |  |
| 28171167 | A | G |  |
| 28171686 | T | G |  |
| 28171793 |  |  | Insertion of a base |
| 28171823 | A | G |  |
| 28172407 | G | A |  |
| 28173603 |  |  | Insertion of a base |
| 28174486 | G | A |  |
| 28177254 | T | A |  |
| 28177803 | G | A |  |
| 28179180 | A | T |  |
| 28180283 |  |  | Insertion of a base |
| 28180612 | G | A |  |
| 28180844 |  |  | Insertion of a base |
| 28181415 | C | T |  |
| 28181604 | C | G |  |
| 28183721 | G | A |  |
| 28183801 | G | A |  |
| 28183820 | A | G |  |
| 28184881 | G | A |  |
| 28184906 | C | T |  |
| 28187171 |  |  | Insertion of a base |
| 28189706 | C | T |  |
| 28190372 | G | C |  |
| 28190795 | T | C |  |
| 28191437 | A | G |  |
| 28192158 | T | C |  |
| 28192280 | TTA | ATT |  |
| 28193755 | G | C |  |
| 28193761 |  |  | Insertion of a base |
| 28193837 |  |  | Insertion of a base |
| 28194315 | C | G |  |
| 28194712 | T | A |  |
| 28194753 | C | A |  |
| 28194753 | C | A |  |
| 28194814 | NONE | T |  |
| 28194850 | G | T |  |
| 28194914 | C | T |  |
| 28194926 | G | A |  |
| 28195022 | C | A |  |
| 28195074 | TAAA | GAAG |  |
| 28195132 | T | G |  |
| 28195630 | T | G |  |
| 28195668 | A | T |  |
| 28195771 | G | A |  |
| 28195796 | G | A |  |
| 28195875 | C | T |  |
| 28195885 | A | G |  |
| 28195917 | GG | AA |  |
| 28195958 | A | G |  |
| 28195970 |  |  | Insertion of a base |
| 28196756 | C | T |  |
| 28198308 | G | A |  |
| 28202239 | T | C |  |
| 28202830 | AT | TA |  |
| 28203826 | A | T |  |
| 28207849 | T | C |  |
| 28208452 | T | C |  |
| 28211743 | NONE | TT |  |
| 28212956 |  |  | Insertion of a base |
| 28213445 | T | C |  |
| 28212556 | NONE | GA |  |
| 28217075 | G | A |  |
| 28219283 | G | A |  |
| 28222799 | G | C |  |
| 28222823 |  |  | Insertion of a base |
| 28222831 | T | A |  |
| 28224607 | A | G |  |
| 28225101 | G | A |  |
| 28228699 |  |  | Insertion of a base |
| 28228882 | A | C |  |
| 28229074 | A | G |  |
| 28229246 | NONE | A |  |
| 28229541 | C | T |  |
| 28230096 | T | C |  |
| 28230434 | NONE | AATAATAAT |  |
| 28230886 | C | T |  |
| 28231963 | A | G |  |
| 28232155 | C | G |  |
| 28232380 | A | G |  |
| 28233551 |  |  | Insertion of a base |
| 28233698 | T | A |  |
| 28235050 | CC | GG |  |
| 28235283 | G | A |  |
| 28235448 | G | T |  |
| 28235508 |  |  | Insertion of a base |
| 28235794 | A | C |  |
| 28236037 |  |  | Insertion of a base |
| 28237219 | G | A |  |
| 28237612 | T | C |  |
| 28237629 | T | A |  |
| 28238054 | AAGGAA | GAGGAC |  |
| 28238316 | A | T |  |
| 28239229 | A | G |  |
| 28239311 | NONE | CTCTC |  |
| 28239423 | G | A |  |
| 28239485 | G | A |  |
| 28239685 | NONE | A |  |
| 28239834 | NONE | TTCATGGACTTTTTTACCCT |  |
| 28240131 |  |  | Insertion of a base |
| 28243063 | T | A |  |
| 28243268 | C | T |  |
| 28244087 | NONE | TG |  |
| 28245022 | T | C |  |
| 28245833 | C | A |  |
| 28245908 |  |  | Insertion of a base |
| 28246890 | T | G |  |
| 28247664 | T | C |  |
| 28247985 | CAA | TCT |  |
| 28248759 | NONE | CAAAACTAGACATCTTTTTTTTTTTTTC |  |
| 28248871 | A | G |  |
| 28249220 |  |  | Insertion of a base |
| 28249691 | C | T |  |
| 28251480 | NONE | TTGTCGTCCT |  |
| 282530002 | T | C |  |
| 28253735 | NONE | A |  |
| 28253999 | G | A |  |
| 28254133 | A | T |  |
| 28254819 | A | G |  |
| 28254894 | C | T |  |
| 28256404 | G | T |  |
| 28256793 | C | T |  |
| 28257080 |  |  | Insertion of a base |
| 28257250 |  |  | Insertion of a base |
| 28257303 | G | C |  |
| 28257719 | A | C |  |
| 28258672 | G | A |  |
| 28259480 | C | T |  |
| 28260071 | T | C |  |
| 28260653 | T | C |  |
| 28262756 | C | T |  |
| 28263112 | T | C |  |
| 28263657 | G | T |  |
| 28263678 |  |  | Insertion of a base |
| 28263835 | G | A |  |
| 28263883 | G | C |  |
| 28263899 | ATC | GTT |  |
| 28263930 | T | C |  |
| 28263950 | NONE | CT |  |
| 28264116 | T | C |  |
| 28264127 | C | T |  |
| 28264231 | C | A |  |
| 28264242 | A | T |  |
| 28264594 |  |  | Insertion of a base |
| 28265452 | NONE | A |  |
| 28265608 | NONE | T |  |
| 28266340 | G | A |  |
| 28267377 | C | A |  |
| 28268360 | NONE | DELETION UNTIL 28268606 |  |
| 28271186 | NONE | TGT |  |
| 28272655 | C | T |  |
| 28273960 | T | C |  |
| 28274361 | C | T |  |
| 28274693 | ACAAACAAAC | CAAACAAACA |  |
| 28277079 | A | G |  |
| 28277123 | C | A |  |
| 28277965 | C | G |  |
| 28278199 | C | T |  |
| 28278644 | T | A |  |
| 28280521 | C | A |  |
| 28281007 | T | C |  |
| 28281043 | NONE | T |  |
| 28281053 | A | G |  |
| 28281070 |  |  | Insertion of a base |
| 28281430 | T | C |  |
| 28282606 | G | A |  |
| 28282768 | T | C |  |
| 28282959 | C | T |  |
| 28283639 | NONE | AC |  |
| 28283649 | T | A |  |
| 28290682 | G | A |  |
| 28293401 |  |  | Insertion of a base |
| 28295078 | NONE | AT |  |
| 28300053 | A | G |  |
| 28300494 | G | A |  |
| 28304214 |  |  | Insertion of a base |
| 28310137 | G | A |  |
| 28310324 | A | C |  |
| 28314421 | A | T |  |
| 28314535 | A | T |  |
| 28315533 | C | G |  |
| 28315707 | NONE | A |  |
| 28316432 | G | A |  |
| 28317250 | T | C |  |
| 28317494 |  |  | Insertion of a base |
| 28317918 | NONE | T |  |
| 28318234 | G | A |  |
| 28319825 | NONE | AAAAA |  |
| 28230755 |  |  | Insertion of a base |
| 28325195 | C | T |  |
| 28328852 | C | T |  |
| 28239475 | C | A |  |
| 28333249 | T | C |  |
| 28335479 | C | T |  |
| 28336595 |  |  | Insertion of a base |
| 28337576 | A | G |  |
| 28338597 | G | A |  |
| 28342376 | T | A |  |
| 28342886 | G | A |  |
| 28343255 | T | A |  |
| 28343312 | A | T |  |
| 28344177 | C | T |  |
| 28344839 | C | T |  |
| 28345605 | NONE | A |  |
| 28348534 | C | T |  |
| 28349070 | C | G |  |
| 28349071 |  |  | Insertion of a base |
| 28349125 | C | T |  |
| 28351012 | G | A |  |
| 28352433 | NONE | G |  |
| 28353258 |  |  | Insertion of a base at CAG area |
| 28353318 | G | C |  |
| 28353791 | C | A |  |
| 28355039 | G | A |  |
| 28356344 |  |  | Insertion of a base |
| 28356806 | T | C |  |
| 28356996 | C | G |  |
| 28358072 | G | A |  |
| 28359251 | G | A |  |
| 28360025 | A | T |  |
| 28361207 | A | G |  |
| 28361455 | T | C |  |
| 28361646 |  |  | Insertion of a base |
| 28361647 | G | A |  |
| 28363358 | TT | AA |  |
| 28364861 | A | G |  |
| 28367477 |  |  | Insertion of a base |
| 28368582 | NONE | TTT |  |
| 28371021 | A | T |  |
| 28371370 | A | G |  |
| 28371724 | A | C |  |
| 28372285 | G | A |  |
| 28372981 | C | T |  |
| 28373062 | C | T |  |
| 28374182 | G | T |  |
| 28374930 | A | G |  |
| 28374964 |  |  | Insertion of a base |
| 28375052 | G | A |  |
| 28375070 | A | G |  |
| 28375227 | T | C |  |
| 28378870 | G | A |  |
| 28379288 | C | T |  |
| 28380868 | A | G |  |
| 28382506 |  |  | Insertion of a base |
| 28393528 |  |  | Insertion of a base |
| 28399655 | A | G |  |
| 28405063 |  |  | Insertion of a base |
| 28428847 | NONE | CT |  |
| 28436740 | A | C |  |
| 28440374 | A | C |  |
| 28440446 |  |  | Insertion of a base |
| 28440717 | T | C |  |
| 28441247 | C | G |  |
| 28441376 | NONE | T |  |
| 28441995 |  |  | Insertion of a base |
| 28443275 | A | G |  |

**Supplemental Table 4: Sequences from PCR amplification of the region of interest in exon 42.** Affected CKCS, normal, and GRMD dog sequences are provided. Nested PCR was performed using outside and inside primers, which were designed to flank the deletion site in the affected CKCS dog. The 330bp canine reference sequence (location chromosome X 27442787..27443187) was cloned into plasmid vector PCR2.1 at the multiple cloning site flanked between the ECOR1 restriction sites. The 7-bp nucleotides at chromosome 27,442,987 “AGCATTA” were deleted in the affected CKCS dog, but can be found in normal and GRMD dogs, as shown in the sequences listed below. In the affected CKCS dog, the deletion occurred between GA (compliment TC). In normal and GRMD, AGCATTA (complement TAATGCT) are present, but are deleted in the affected CKCS dog. Sequence of vector PCR2.1. GAATTC = restriction site ECOR1. Outside primers, sense and anti-sense. Inside primers, sense and anti-sense.

| CKCS (5’🡪3’)  TGTTTCGCCTACCTATAGGGCGAATATGGGCCCTCATAGTTGCATTGCTCGAGCGGCCGCCAGTGTGATGGATATCTGCAGAATTCGGCTTGTGGTTTAGGAATTCCACATGTACGTTAAAATGCCTTGGTGTGTACCTCTAAGTCAATCATTGTGGTACTGCGAATGATCAAGAAGAACAGCTTGTAAAAATCTCATAAAAATGCTTTGCTTTTACCTTCAAGGATTCCTCTTGTTTAAAGAGATCTTCAAAATCTTGAGCACAAAGGTCGGGAGAAGCTCTTCCACTTCTGATAGGGCTTGTGAGACATGAGTGATCTCAGTCAGGTAAGTAGAAGGCACATAAGAAATTTCCAAAGGCATGTCTTCAGTCATCGCCACCACTGACTCTTCATGGACAGTGTGCTGGTATAGACAAAGCCGAATTCCAGCACACTGGCGGCCGTTACTAGTGGATCCGAGCTCGGTACCAAGCTTGGCGTAATCATGGTCATAGCTGTTTCCTGTGTGAATTGTTATCCGCTCACAATTCCACACAACATACGAGCCGGAAGCATAAAGTGTAAAGCCTGGGGTGCCTAATGAGTGAGCTAACTCACATTAATTGCGTTGCGCTCACTGCCCGCTTTCCAGTCGGGAAACCTGTCGTGCCAGCTGCATTAATGAATCGGCCAACGCGCGGGGAGAGGCGGGTTTGCGTATTGGGCGCTCTTCCGCTTTCCTCGCTCACTGACTCGCTGCGCTCGGTCGTTCGGCTGCGCGAGCGGTATCAAGCTCAATCAAAGGCGGTAATACGGGTTATTCCAACAAGGAATCAGGGATAACGCCAGGGAAAAGAACATGTGAGCCAAAAGGCCAGCCTAAGCCCAGGAACCGTAAAAAGGCGCGATGCTTGGCGATTTTACATAAGCTCCGCCCCCTGAGCCAGCATCACAAAATCGAGGCCTCAAGTCGAAGGTTGCCGAAAATCCGAC |
| --- |
| Normal (3’🡪5’)  AAGTCGACTATATAGGGCGAATTGGGCCCTCAAGATGCATGCTCGAGCGGCCGCCAGTGTGATGGATATCTGCAGAATTCGGCTTTGTCTATACCAGCACACTGTCCATGAAGAGTCAGTGGTGGCGATGACTGAAGACATGCCTTTGGAAATTTCTTATGTGCCTTCTACTTACCTGACTGAGATCACTCATGTCTCACAAGCCCTATCAGAAGTGGAAGAGCTTCTTAATGCTCCCGACCTTTGTGCTCAAGATTTTGAAGATCTCTTTAAACAAGAGGAATCCTTGAAGGTAAAAGCAAAGCATTTTTATGAGATTTTTACAAGCTGTTCTTCTTGATCATTCGCAGTACCACAACGATTGACTTAGAGGTACACACCAAGGCATTTTAACGTACATGTGGAATTCCTAAACCACAAGCCGAATTCCAGCACACTGGCGGCCGTTACTAGTGGATCCGAGCTCGGTACCAAGCTTGGCGTAATCATGGTCATAGCTGTTTCCTGTGTGAAATTGTTATCCGCTCACAATTCCACACAACATACGAGCCGGAAGCATAAAGTGTAAAGCCTGGGGTGCCTAATGAGTGAGCTAACTCACATTAATTGCGTTGCGCTCACTGCCCGCTTTCCAGTCGGGAAACCTGTCGTGCCAGCTGCATTAATGAATCGGCCAACGCGCGGGGAGAGGCGGTTTGCGTATTGGGCGCTCTTCCGCTTTCCTCGCTCACTGACTCGCTGCGCTCGGTCGTTCGGCTGCGGCGAGCGGTATCAGCTCACTCAAAGGCGTAATACGGTTATCCACAGGATCAGGGATAACCGCAGGAAAAGAAACAATTGTGGAGGCAAAGCAGCAAAGCAGACCGTAAAAGCCCGCGTTGCTTGGGCGTTTTCCATAAGGCCTCCCGGCCCCCTTGGAGCCGAGCACATCCCACAGAT |
| GRMD (5’🡪3’)  TGTGACGCCTACTATAGGGCGAATTGGGCCTCAAGATGCACGCTCGAGCGGCCGCCAGTGTGATGGATATCTGCAGAATTCGGCTTGTGGTTTAGGAATTCCACATGTACGTTAAAATGCCTTGGTGTGTACCTCTAAGTCAATCGTTGTGGTACTGCGAATGATCAAGAAGAACAGCTTGTAAAAATCTCATAAAAATGCTTTGCTTTTACCTTCAAGGATTCCTCTTGTTTAAAGAGATCTTCAAAATCTTGAGCACAAAGGTCGGGAGCATTAAGAAGCTCTTCCACTTCTGATAGGGCTTGTGAGACATGAGTGATCTCAGTCAGGTAAGTAGAAGGCACATAAGAAATTTCCAAAGGCATGTCTTCAGTCATCGCCACCACTGACTCTTCATGGACAGTGTGCTGGTATAGACAAAGCCGAATTCCAGCACACTGGCGGCCGTTACTAGTGGATCCGAGCTCGGTACCAAGCTTGGCGTAATCATGGTCATAGCTGTTTCCTGTGTGAAATTGTTATCCGCTCACAATTCCACACAACATACGAGCCGGAAGCATAAAGTGTAAAGCCTGGGGTGCCTAATGAGTGAGCTAACTCACATTAATTGCGTTGCGCTCACTGCCCGCTTTCCAGTCGGGAAACCTGTCGTGCCAGCTGCATTAATGAAATCGGCCAACGCGCGGGGGAGAGGGCGGTTTGGCGTTATTTGGGGGCGCTTCTTTCCGCTTTCCTTCCGCCTCCACTGGACTCGCTGGCGCTCGGGTTCGTTTCGGCTGGCGGCGAGGCGGGTATTCAGCCTCACCTCCAAAGGGCGGTAATACCGGGTTTATCCAACAGAATCAGGGGAATAACCGCCAGGAAAGAAAACAATTGTGGTGGAGCCCAAAAGGCCAGTCAAAAGGTCTAGGAATCCGATTAAAAGCCCCGCGTCGCTGACGTATTCCCAATAGGCTTCGGACCCCT |
